# Direct Regulation of Cytochrome *c* Oxidase by Calcium Ions

**DOI:** 10.1371/journal.pone.0074436

**Published:** 2013-09-10

**Authors:** Tatiana Vygodina, Anna Kirichenko, Alexander A. Konstantinov

**Affiliations:** A. N. Belozersky Institute of Physico-Chemical Biology, M. V. Lomonosov Moscow State University, Moscow, Russia; University of Saskatchewan, Canada

## Abstract

Cytochrome *c* oxidase from bovine heart binds Ca^2+^ reversibly at a specific Cation Binding Site located near the outer face of the mitochondrial membrane. Ca^2+^ shifts the absorption spectrum of heme *a,* which allowed previously to determine the kinetics and equilibrium characteristics of the binding. However, no effect of Ca^2+^ on the functional characteristics of cytochrome oxidase was revealed earlier. Here we report that Ca^2+^ inhibits cytochrome oxidase activity of isolated bovine heart enzyme by 50–60% with K_i_ of ∼1 µM, close to K_d_ of calcium binding with the oxidase determined spectrophotometrically. The inhibition is observed only at low, but physiologically relevant, turnover rates of the enzyme (∼10 s^−1^ or less). No inhibitory effect of Ca^2+^ is observed under conventional conditions of cytochrome *c* oxidase activity assays (turnover number >100 s^−1^ at pH 8), which may explain why the effect was not noticed earlier. The inhibition is specific for Ca^2+^ and is reversed by EGTA. Na^+^ ions that compete with Ca^2+^ for binding with the Cation Binding Site, do not affect significantly activity of the enzyme but counteract the inhibitory effect of Ca^2+^. The Ca^2+^-induced inhibition of cytochrome *c* oxidase is observed also with the uncoupled mitochondria from several rat tissues. At the same time, calcium ions do not inhibit activity of the homologous bacterial cytochrome oxidases. Possible mechanisms of the inhibition are discussed as well as potential physiological role of Ca^2+^ binding with cytochrome oxidase. Ca^2+^- binding at the Cation Binding Site is proposed to inhibit proton-transfer through the exit part of the proton conducting pathway H in the mammalian oxidases.

## Introduction

Cytochrome *c* oxidase (COX) is a terminal enzyme of the mitochondrial and bacterial respiratory chains. The enzyme catalyses reduction of molecular oxygen to water coupled to translocation of protons across the coupling membrane [1]–[3]:

4 cyt *c* ^2+^ + O_2_ + 8H^+^_inside_ = 4 cyt *c* ^3+^ + 2H_2_O + 4H^+^_outside_.

The electron transfer in the oxidase is mediated by four metal redox centers: two A-type hemes, low-spin *a* and high-spin *a*_3_, and two copper centers, a binuclear Cu_A_ and a mononuclear Cu_B_. The high-spin heme *a*_3_ iron and Cu_B_ are located within ∼5 A from each other and form a di-nuclear site of oxygen reduction to water. The sequence of electron transfer through the enzyme is described by a scheme.

cyt *c* → Cu_A_ → heme *a* → heme *a*_3_/Cu_B_ → O_2_.

In addition to the redox centers, cytochrome oxidases from mitochondria and many bacteria contain non-redox metal ions, revealed by chemical analysis [4] and identified later on in the crystal structure of the enzyme [5], [6]. First, there is Mg^2+^ (or Mn^2+^) ion which holds together subunits I and II and may be part of the exit pathway for the pumped protons and for water formed in the active site [7]–[9]. Second, there is a zinc finger in subunit Vb of bovine heart oxidase [5], [6], function of which is not known yet. Third, a novel metal cation binding site (CBS) was resolved in the 3D structure of COX from both mitochondria and bacteria (refs. [10], [11] and PDB entry 1M56) that can harbour Ca^2+^ or Na^+^ ion.

Reversible binding of Ca^2+^ with the mitochondrial COX was described originally by Wikstrom and Saari [12], who reported a blue shift of the reduced heme *a* α-absorption band induced by EDTA and showed that the effect was due to reversal of a red shift induced by adventitious calcium ions acting from the outer side of the mitochondrial membrane. The specific CBS was identified later on in the crystal structures of the A1-class [13] cytochrome oxidases from two bacteria (*P. denitrificans* [10] and *R.sphaeroides* (PDB entry 1M56)) and bovine heart [11].

**Figure 1:**
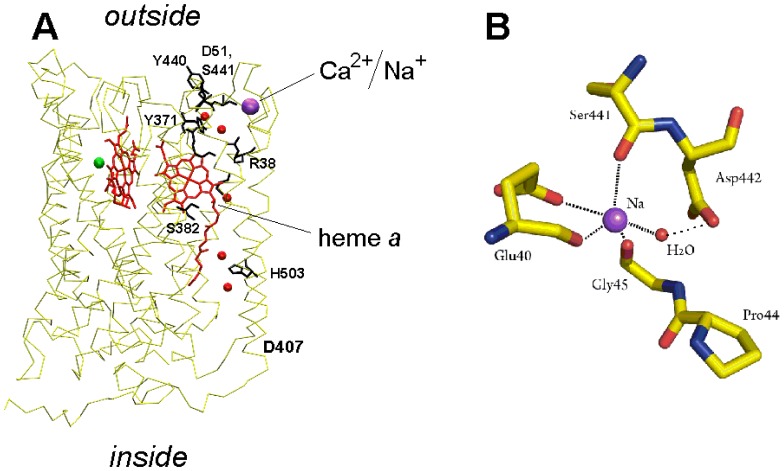
The Cation Binding Site in cytochrome c oxidase. (**A**) Location of the Cation Binding Site in subunit I of bovine enzyme and its relation to the proposed proton conducting pathway H. Components of the H-pathway are depicted as orange spheres (fixed water molecules) and black sticks (amino acid residues, A-propionate and carbonyl groups of heme *a*). Enlarged picture of the exit part of the H-channel is shown in Figure 8. (**B**) Coordination sphere of the bound cation in bovine oxidase. Based on the PDB 1V55 structure.

As shown in **Figure 1A**, the site is located at the very periphery of subunit I facing the outer side of the membrane, within ca. 18A from the Fe ion of heme *a*. In the bacterial oxidases, the X-ray structure and chemical analysis reveal tightly bound Ca^2+^ at the site [14], [15]. The cation cannot be removed by calcium chelators. Accordingly, addition of Ca^2+^ does not induce spectral shift of heme *a* in COX from *R. sphaeroides* [16] or *P. denitrificans* [14]. However, mutations in some of the residues in coordination sphere of Ca^2+^ in COX from *P.denitrificans* [14], [17], [18] or *R. sphaeroides* [15] result in release of the tightly bound cation and in reversible binding of Ca^2+^ with the bacterial enzyme, making the bacterial oxidases a useful model for the studies of CBS in the mammalian oxidase.

Originally reported to be absolutely specific for Ca^2+^ and proton [19], the CBS was shown later on to bind also Na^+^ as revealed by competition of the latter with Ca^2+^ [16], [20]. Furthermore, the published crystal structure of the mitochondrial enzyme resolved Na^+^ rather than Ca^2+^ bound at the site (**Figure 1B**, ref. [11]). This is not surprising since the crystals were obtained at ∼10 mM Na^+^ in the buffer, that is well above K_d_ for Na^+^ binding with the site (ca. 10^−3^ M [16]). Very recently, competing binding of Ca^2+^ and Na^+^ with CBS of bovine COX was confirmed by FTIR spectroscopy [21].

As Ca^2+^ brings about a red shift of heme *a* absorption spectrum in both the reduced and oxidized forms, it is easy to monitor binding of the cation with the enzyme at different oxidation states. Equilibrium and kinetic parameters of the binding have been studied in considerable detail for the mitochondrial and mutant bacterial oxidases [14]–[20]. Initially, K_d_ for Ca^2+^ binding with the mitochondrial oxidase was reported to be 20–30 µM [12], [19]. The values were so much higher than typical concentrations of free Ca^2+^ in the cytoplasm (∼10^−7^ M [22], [23]) that calcium binding with COX was not considered to be of physiological relevance and did not receive much attention. However, subsequent studies with the use of Ca^2+^-buffering systems determined much lower K_d_ value of ∼1 µM [16], [18], which is well in the range of cytoplasmic Ca^2+^ concentrations attained during the Ca^2+^ spikes induced by Ca^2+^ efflux from the cisterns of endoplasmic reticulum [22], [23].

Ca^2+^ is a ubiquitous intracellular signal transduction messenger that regulates a vast number of processes in the cell [22], [23]. In particular, Ca^2+^ is known to enhance oxidative phosphorylation in mitochondria (reviewed, [24], [25]) by stimulating activity of several Krebs cycle dehydrogenases in the mitochondrial matrix and also by activating several mitochondrial substrate transporters [26], [27]. High affinity binding of Ca^2+^ with COX at a specific site at the outer face of the inner mitochondrial membrane implied that cytoplasmic Ca^2+^ could be a physiological effector of the mitochondrial COX [15], [16], [18]. Disappointingly, previous attempts to reveal any effect of Ca^2+^ on the functional characteristics of COX were not successful. In this paper, we describe inhibition of COX induced by Ca^2+^ binding at the CBS. The reasons why this effect was not noticed earlier as well as possible mechanisms and physiological role of the Ca^2+^-induced inhibition are discussed. The data were presented at the 2010 EBEC Meeting at Warsaw [28], [29].

## Materials and Methods

### Chemicals

Sodium dithionite, CaCl_2_, choline chloride (C-1879, >99% ), carbonyl cyanide *m*-chlorophenyl hydrazone (CCCP), cyclosporine A, nagarse, cytochrome *c* type III, *N,N,N’,N’* – tetrametyl-*p*-phenylenediamine (TMPD), potassium salts of ferrocyanide and ferricyanide, and calcium buffers: ethylene-bis(oxyethylenenitrilo)tetraacetic acid (EGTA), nitrilotriacetic acid (NTA), 1,2-bis(2-aminophenoxy)-ethane- *N,N,N’,N’*- tetraacetic acid (BAPTA) and *N*-(2-hydroxyethyl)ethylenediamine- *N,N’,N’*-triacetic acid (HEDTA) were from Sigma-Aldrich. pH buffers, sodium chloride and magnesium sulfate were purchased from Amresco. Dodecylmaltoside (DM) “SOL-GRADE” was from Anatrace.

### Preparations

“Fast” COX was purified from bovine heart mitochondria using a modified protocol by Fowler et al. [30]. Bovine hearts were purchased from slaughterhouse “OOO Pushkinsky Meet House” (23 Sokolovstkaya str., Pushkino, Moscow region). The hearts were sold in agreement with the A.N.Belozersky Institute request letter under the condition that they may not be used for commercial purposes, but for scientific research purposes only. COX from *R. sphaeroides* was purified from bacterial membranes (a kind gift from Dr. R. Gennis laboratory at UIUC, IL) on a column with Ni^2+^-NTA Sepharose (Qiagen) [31]. A sample of D477A mutant COX from *P.denitrificans* was kindly provided by Dr. Anne Puustinen (Helsinki Bioenergetics Group, University of Helsinki). Concentration of COX was determined from the “dithionite-reduced *minus* oxidized” difference absorption spectra using Δε_605–630_ of 27 mM^−1^cm^−1^. Mitochondria from rat tissues were isolated from outbred white male rats by conventional methods as used in this laboratory [32], [33] with additional protease treatment (Nagarse) [34] to disrupt the outer mitochondrial membrane and remove permeability barrier for added cytochrome *c* and calcium ions. After the treatment, mitochondria were washed thoroughly to remove Nagarse. Animal protocols were approved by the Institutional Review Board. Handling of the animals and experimental procedures were conducted in accordance with the international guidelines for animal care and use and were approved by the Institutional Ethics Committee of A.N. Belozersky Institute of Physico-Chemical Biology at Moscow State University.

### Assays

Experiments with the isolated COX were performed in a basic medium containing 50 mM Tris/MES pH 8.0–8.2, 0.05% dodecyl maltoside, choline chloride (50 mM or higher as indicated), and also 100 µM EGTA to bind adventitious calcium and strip off the bound calcium from the mitochondrial oxidase. In order to slow down reaction of cytochrome *c* with COX, in some experiments we had to increase the ionic strength of the reaction mixtures. To this end, choline chloride was chosen as the main salt component of the reaction buffers because, in our experience, this salt as available from Sigma-Aldrich (C-1879) proved to have very low contamination with Na^+^. Concentration of sodium ions in the media has to be minimized because Na^+^ competes with Ca^2+^ for binding with COX [16], [20]. In experiments with mitochondria, the basic buffer contained 0.25 M sucrose, 0.4 M choline chloride, 50 mM Tris/MES or Tris/HEPES pH 8, 100 µM EGTA and also 1 µM cyclosporine A and 1 µM the uncoupler, CCCP. Concentration of free calcium in the buffers at given concentrations of the added CaCl_2_, Ca^2+^-buffering chelators (EGTA, HEDTA, BAPTA, NTA) and other specific conditions was calculated as earlier [18] with a free software “WinMAXC, v.2.05”.

Spectrophotometric measurements were made in a Varian Cary300Bio or an SLM-Aminco 2000C instrument in 10 mm optical pathway cells at 25°C. Oxidation of ferrocytochrome *c* was measured in a dual-wavelength mode at 550 nm vs a reference wavelength at 535 or 540 nm. Turnover numbers (TN) for COX (e.g., 10 s^−1^) are expressed in electrons per second per cytochrome oxidase monomer, if not stated otherwise. The Ca^2+^-induced red shift of heme *a* absorption spectrum was measured in the SLM-Aminco 2000C spectrophotometer operated in a split beam mode. COX was pre-reduced by 5 mM ascorbate and 100 µM TMPD in the presence of 5 mM KCN that resulted in complete reduction of heme *a*. The difference spectra induced in the α-band of heme *a* by increasing concentrations of CaCl_2_ in the presence of Ca^2+^-buffers were recorded. The amplitude of the derivative-shaped difference spectra at 613 nm minus 599–600 nm [16], [19] was plotted vs the concentration of free Ca^2+^. The data were processed with OriginLab 7e software package (Microcal). The figures with the crystal structure of COX domains were prepared with the aid of PyMOL software.

## Results

Our conjecture was that the putative regulatory effect of Ca^2+^ on COX activity, if in existence, would be more pronounced under the conditions of the enzyme turnover close to those in the respiring mitochondria. COX turnover in the respiring mitochondria (cf. [35], [36]) differs from the standard cytochrome oxidase activity assays by at least two important parameters. First, in the standard assays, turnover rate of cytochrome oxidase is close to V_max_ (for bovine oxidase, ca. 200–600 s^−1^ depending on pH), whereas in the mitochondria respiring on succinate or NADH-dependent substrates, COX turns over much slower, ca. 10 s^−1^ or less, even in the fully uncoupled state. Second, in the standard assays, cytochrome *c* is kept almost fully reduced (e.g., by excess ascorbate and TMPD) and its redox potential, E_h_, is much lower than E_m_, whereas in mitochondria respiring with succinate or NADH-dependent susbstrates, cytochrome *c* is typically less than half-reduced in the steady-state [35], [36]. Therefore, we have searched for effect of Ca^2+^ on COX activity at slow turnover rates of the enzyme and at high redox potential of the electron donors.

### Inhibition of Purified COX by Ca^2+^

**Figure 2:**
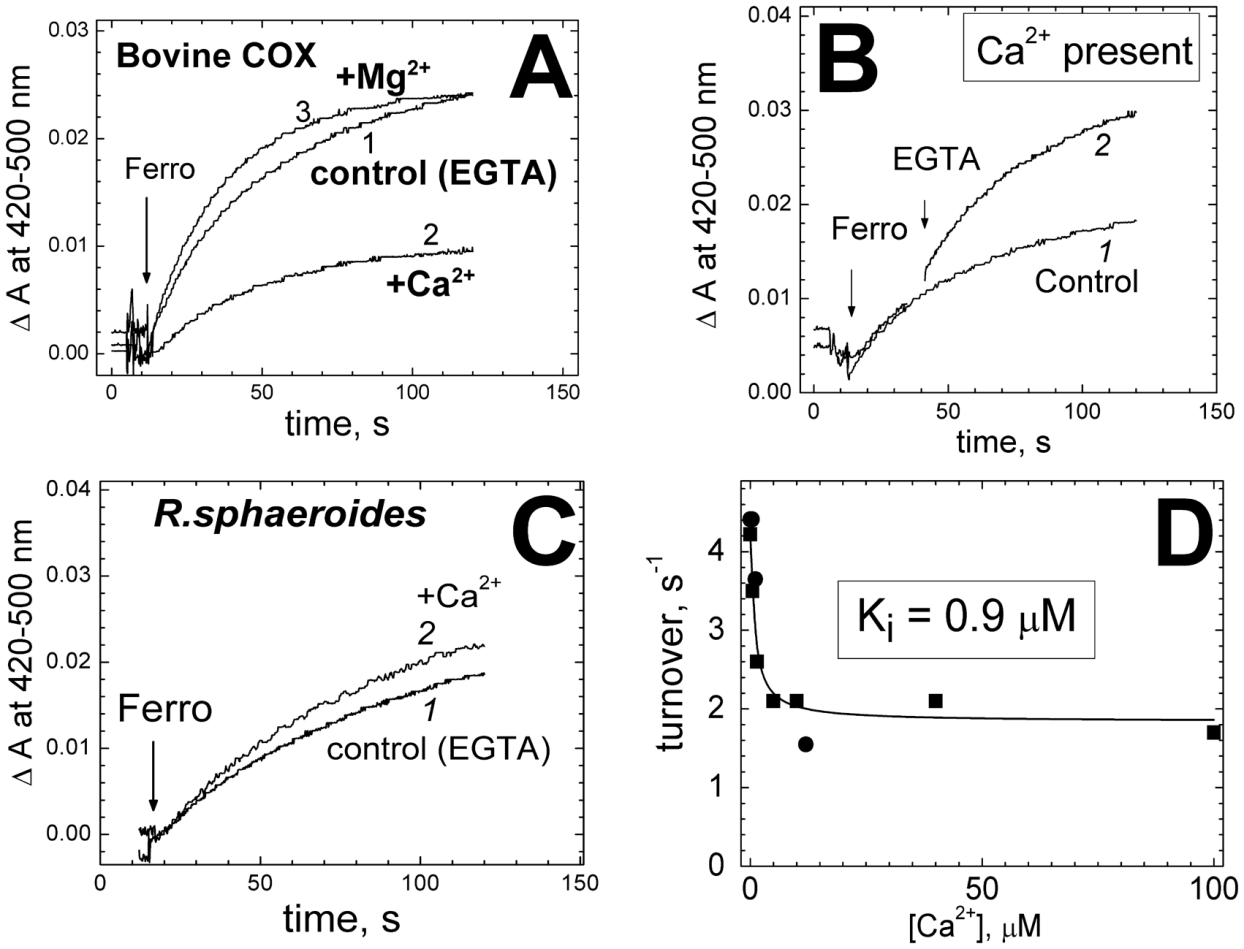
Ca^2+^ ions inhibit cytochrome *c-* catalyzed oxidation of ferrocyanide by cytochrome oxidase. (**A**) 0.3 µM bovine COX in the basic medium (100 µM EGTA present) with 40 nM cytochrome *c*. Where indicated, 1 mM ferrocyanide (ferro) was added, and its oxidation to ferricyanide was followed spectrophotometrically at 420 nm vs the 500 nm reference. Trace *1*, control recording; trace *2*, +200 µM CaCl_2_ (100 µM excess over EGTA); trace *3*, +0.4 mM Mg^2+^ (300 µM excess over EGTA). (**B**) Conditions, as in panel A, trace *2* (100 µM excess of CaCl_2_ over EGTA). In trace *2*, 300 µM EGTA has been added. (**C**) 0.2 µM wild-type COX from *R.sphaeroides*. Trace *1*, control (100 µM EGTA). Trace *2*, with 200 µM CaCl_2_. (**D**) Concentration dependence of the inhibition. Initial rate of ferrocyanide oxidation by COX (0.3 µM) is plotted vs concentration of free Ca^2+^ buffered with 1 mM NTA (squares) or 1 mM BAPTA (circles). The curve corresponds to K_i_ of 0.9 µM and maximal inhibition of 60%.

In the first series of experiments, slow aerobic turnover of purified bovine COX with ferrocyanide as the high-potential electron donor was studied in the presence of catalytic amount (40 nM) of cytochrome *c* [37]. The reaction decelerates with time mainly due to accumulation of ferricyanide raising further the redox potential, E_h_, of the donor. As shown in **Figure 2A**, the cytochrome *c*-catalyzed oxidation of ferrocyanide by COX is significantly inhibited by Ca^2+^ (trace *2*) but not by Mg^2+^ (trace *3*), and the inhibition is reversed by EGTA (**Figure 2B**). The same results were obtained in the control experiments in which 50 mM choline chloride in the reaction buffer was replaced by 50 mM KCl. No inhibition, but rather slight stimulation is observed with the wild type COX from *R. sphaeroides* (**Figure 2C**) containing tightly-bound Ca^2+^ at the site. A small activating effect on ferrocyanide oxidation by the bacterial oxidase was also observed with Mg^2+^ (not shown) and is most likely due to stimulation of direct interaction of ferrocyanide anion with the negatively charged electron entry site of the enzyme by divalent cations [38] (cf. the slight stimulation of the reaction by Mg^2+^ in **Figure 2A**). The inhibition titrates with Ca^2+^ according to a hyperbolic curve with K_i_ of 0.9 µM and maximal inhibition of ∼60% **(** **Figure 2D** **).**

**Figure 3:**
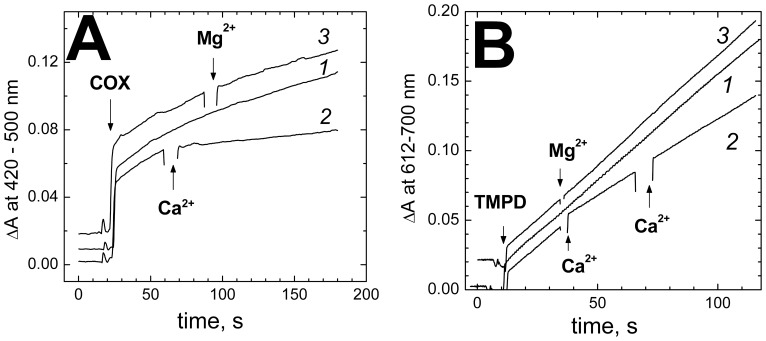
Ca^2+^ inhibits aerobic oxidation of artificial electron donors by COX. (**A**) *Oxidation of ferrocyanide.* 0.2 mM ferrocyanide in the basic buffer pH 8.2, supplemented with 30 µg/ml of poly-L-lysine to stimulate reaction of COX with ferrocyanide anion [38]. Reaction is initiated by addition of 0.4 µM bovine COX and accumulation of ferricyanide is followed at 420 nm vs the 500 nm reference. Trace *1*, control recording with no other additions; trace *2*, 200 µM CaCl_2_ added where indicated; trace *3*, 400 µM MgSO_4_ added instead of CaCl_2_. The initial upward jump of the traces is due to absorption of the added COX. (**B**) *Oxidation of N,N,N’,N’ – tetrametyl-*p*-phenylenediamine.* 0.15 µM bovine COX in the basic buffer. Where indicated, 0.1 mM reduced TMPD is added, and its oxidation to Wurster’s Blue is followed spectrophotometrically at 612 nm vs the 700 nm reference. Trace *1*, control recording with no additions; trace *2,* 200 µM CaCl_2_ added where indicated, note that the second addition does not induce any further inhibition; trace *3*, 200 µM Mg^2+^ added where indicated. The kinetics curves in the panels A and B are displaced arbitrarily on the ordinate axis for clarity.

The Ca^2+^-induced inhibition of COX is also observed with artificial electron donors, such as TMPD and ferrocyanide, in the absence of cytochrome *c* (**Figure 3AB**). The inhibition observed with TMPD (37±4%, 4 experiments) seems to be somewhat less than measured with the other electron donors tested (50–60%). However, it will increase to ∼50% if corrected for ca. 25% of KCN-insensitive oxidation of TMPD in the presence of COX.

**Figure 4:**
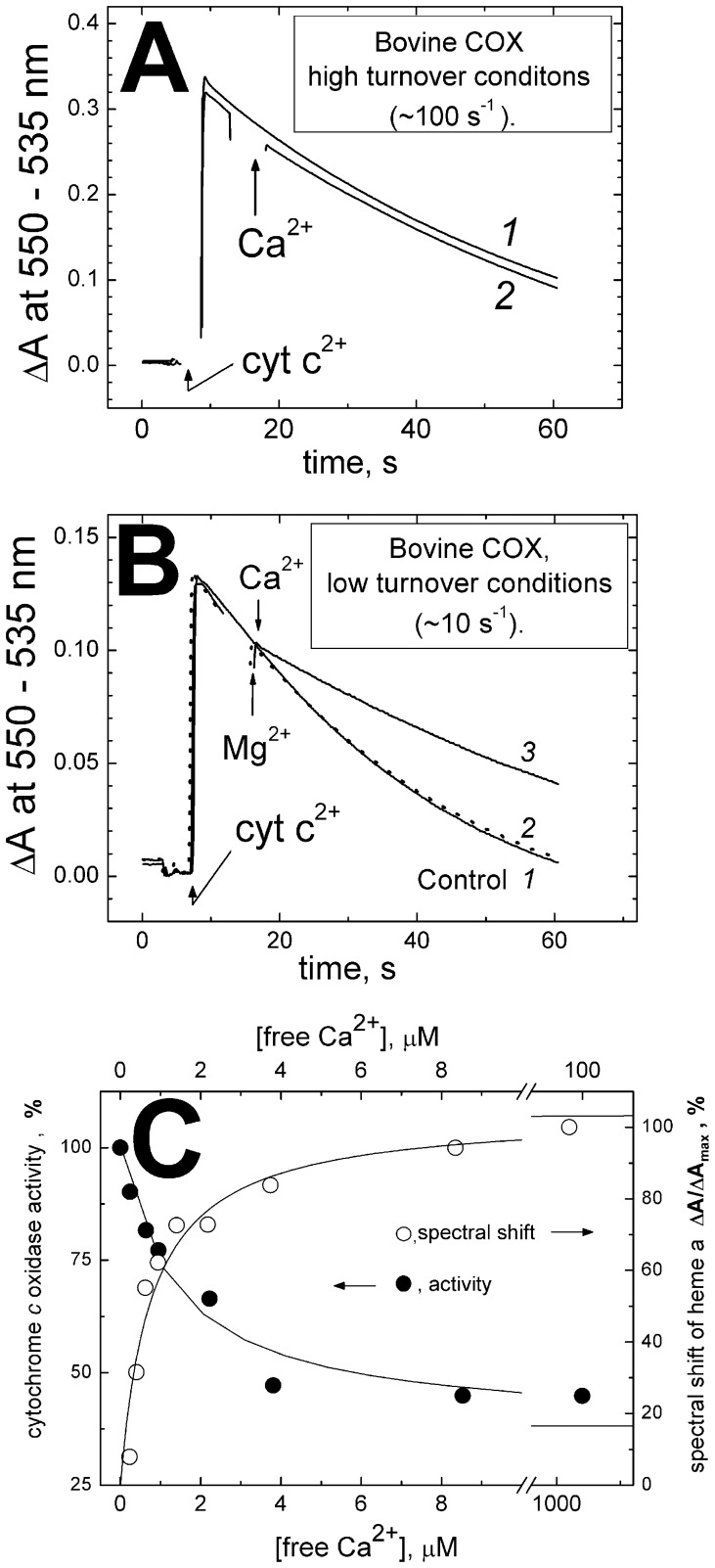
Inhibition of the ferrocytochrome *c* oxidase activity of bovine COX by Ca^2+^ ions. (**A**) *High-turnover conditions.* 4 nM COX in the basic medium (with 50 mM choline chloride and 100 µM EGTA). 18 µM reduced cytochrome *c* is added and its subsequent oxidation is followed spectrophotometrically at 550 nm vs the 535 nm reference. Trace *1*, control recording; trace *2*, 200 µM CaCl_2_ added where indicated. (**B**) *Low-turnover conditions.* Conditions as in (**A**), but choline chloride concentration increased to 0.5 M and 18 µM oxidized cytochrome *c* present in the buffer; COX concentration raised to 20 nM. Trace *1* (solid line), control recording; traces *2,3*: where indicated, 0.4 mM MgCl_2_ (dashed line) or 200 µM CaCl_2_ (solid line) are added. (**C**) Titration of the Ca^2+^-induced inhibition of COX activity and of the red shift of heme *a* spectrum. Cytochrome *c* ^2+^ oxidation was measured as in Panel **B**, trace *1* at different concentrations of free calcium buffered with 5 mM HEDTA. The initial rates were used to build the plot. Spectral shift measurements (see Materials and Methods) were made in the basic buffer with 2 µM COX. Concentration of free Ca^2+^ was buffered with 5 mM HEDTA. The points are fitted by the curves: maximal inhibition, 63±5%, K_i_ = 1.4±0.4 µM; the spectral shift, ΔA_max_ = 103% of the highest experimentally observed ΔA value taken as 100%; K_d_ = 0.77±0.19 µM.

In the second set of experiments, effect of Ca^2+^ on oxidation of excess ferrocytochrome *c* by COX was studied in the absence of artificial redox compounds. A representative experiment is shown in **Figure 4**. Where indicated (“low turnover conditions”), the reaction rate was attenuated by increasing the ionic strength with high concentration of choline chloride and by including ferric cytochrome *c* in the medium. In a standard assay (“high turnover conditions”), no effect of Ca^2+^ is observed (**Figure 4A** **)**. However, if the conditions are set so as to decrease the rate of COX activity to ∼5–10 s^−1^ (**Figure 4B**), addition of Ca^2+^ brings about ca. 2-fold inhibition of cytochrome *c* ^2+^ oxidation (**Figure 4B** **,** trace *3*). The inhibitory effect of Ca^2+^ at turnover rates below ∼10 s^−1^ has been documented for several large scale preparations of bovine CcO with but minor differences in the extent of inhibition among the preparations. In replicate experiments with the same sample of the enzyme at initial turnover rate of 6.2±0.05 s^−1^, the inhibition induced by 100 µM free calcium gives a value of 58±2% (15 experiments). No such effect is induced by Mg^2+^ (**Figure 4B**, trace *2*). Similarly to the results shown in **Figure 2**, the inhibitory effect of Ca^2+^ on ferrocytochrome *c* oxidation was reversed and prevented by excess EGTA and was not observed with COX from *R. sphaeroides* (data not included). The inhibitory effect of Ca^2+^ titrates according to a hyperbolic curve with the maximal inhibition of 50–60% and *K*_i_ of ∼1.4 µM (**Figure 4C** **,** filled circles) in good agreement with the data on ferrocyanide oxidation (**Figure 2D**). The inhibition curve matches well induction of the red shift of heme *a* (**Figure 4C**, open circles).

**Figure 5:**
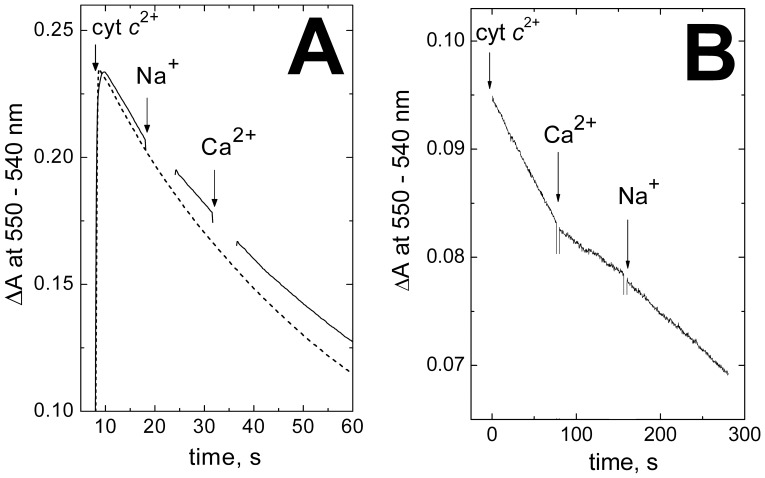
Na^+^ ions counteract the Ca^2+^ -induced inhibition of cytochrome *c* oxidase activity of bovine COX. (**A**) Bovine COX (30 nM) in the basic medium with 0.4 M choline chloride and 15 µM oxidized cytochrome *c*. The reaction is initiated by addition of 15 µM ferrocytochrome *c* and oxidation of *c* ^2+^ is followed in a dual-wavelength mode at 550 nm vs the 540 nm reference wavelength. Dashed line, control recording (no additions). Solid line, 50 mM NaCl and 200 µM CaCl_2_ (100 µM excess over EGTA) are added where indicated. (**B**) Bovine COX (1.3 nM) in the basic medium with 0.4 M choline chloride and with no oxidized cytochrome *c*. Reaction is initiated by addition of 6 µM ferrocytochrome *c*. Other additions, as in (**A**).

Na^+^ competes with Ca^2+^ for binding with COX [16], [18], [20], but does not affect significantly the activity of the enzyme **(** **Figure 5A** **)**. For instance, under the experimental conditions at which 100 µM free Ca^2+^ inhibits the reaction by 58±2%, addition of 50 mM Na^+^ (that is 12-fold the K_d_ value [16]), decreases the rate of ferrocytochrome *c* oxidation by 9±5% (5 experiments). At the same time, 50 mM Na^+^ largely prevents inhibition of the activity by Ca^2+^ (**Figure 5A**), and partial release of the inhibition imposed by Ca^2+^ could be observed (**Figure 5B** **)**. These data are coherent with the observations that Na^+^ does not induce red shift of heme *a* [16], [20] and does not affect E_m_ of heme *a* [39] but counteracts induction of these effects by Ca^2+^ ions.

**Figure 6:**
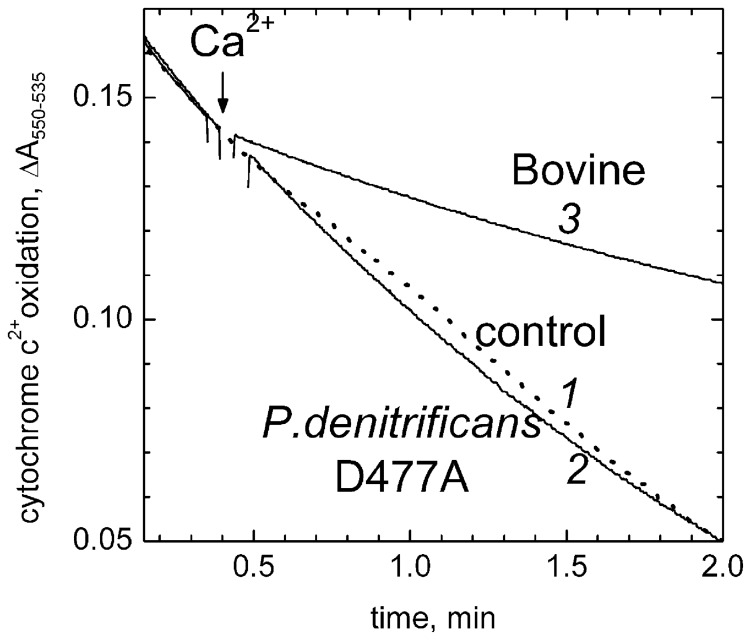
Ca^2+^ does not inhibit cytochrome *c* ^2+^ oxidase activity of D477A mutant COX from *P.denitrificans*. D477A mutant COX (1.5 nM) in the basic medium with 0.4 M choline chloride. Oxidation of 10 µM reduced cytochrome *c* is followed spectrophotometrically in a dual-wavelength mode at 550 nm vs the 535 nm reference. Trace *1* (dotted line), control recording with no calcium added; trace *2*, 200 µM CaCl_2_ added where indicated. Trace *3*, the same conditions as in trace *2* but with 2.5 nM bovine heart COX.

Mutations in the conserved aspartate in the CBS of the bacterial oxidases from *P. denitrificans* or *R.sphaeroides* (D477A_Pd_ or D485A_Rs_, homologous to D442 in bovine enzyme, cf. **Figure 1B**) release the tightly-bound Ca^2+^ from the CBS of the bacterial enzymes and allow to observe reversible binding of exogenous Ca^2+^ and Na^+^ with the site [14], [15], [17], [18]. Interestingly, we found that Ca^2+^ does not affect ferrocytochrome *c* oxidation by either D485A mutant COX from *R. sphaeroides* (data not included) or D477A mutant COX from *P.denitrificans* (**Figure 6**). Thus, the inhibitory effect of calcium ions may be specific for the mammalian oxidase.

### Inhibition of the Cytochrome c^2+^ Oxidase Activity in Mitochondria

The inhibitory effect of Ca^2+^ was further confirmed with COX in its native surroundings, i.e. in the mitochondrial membrane. To this end, we have measured spectrophotometrically ferrocytochrome *c* oxidase activity of mitochondria from several rat tissues. The outer membrane of the mitochondria was disrupted by Nagarse treatment [34] to make it permeable to cytochrome *c*. The Nagarse-treated mitochondria still showed respiratory control, therefore the experiments were carried out in the presence of CCCP, the uncoupler, and 1 µM cyclosporin A to preclude Δψ-driven Ca^2+^ accumulation in the mitochondrial matrix, swelling of the mitochondria and pore opening.

**Figure 7:**
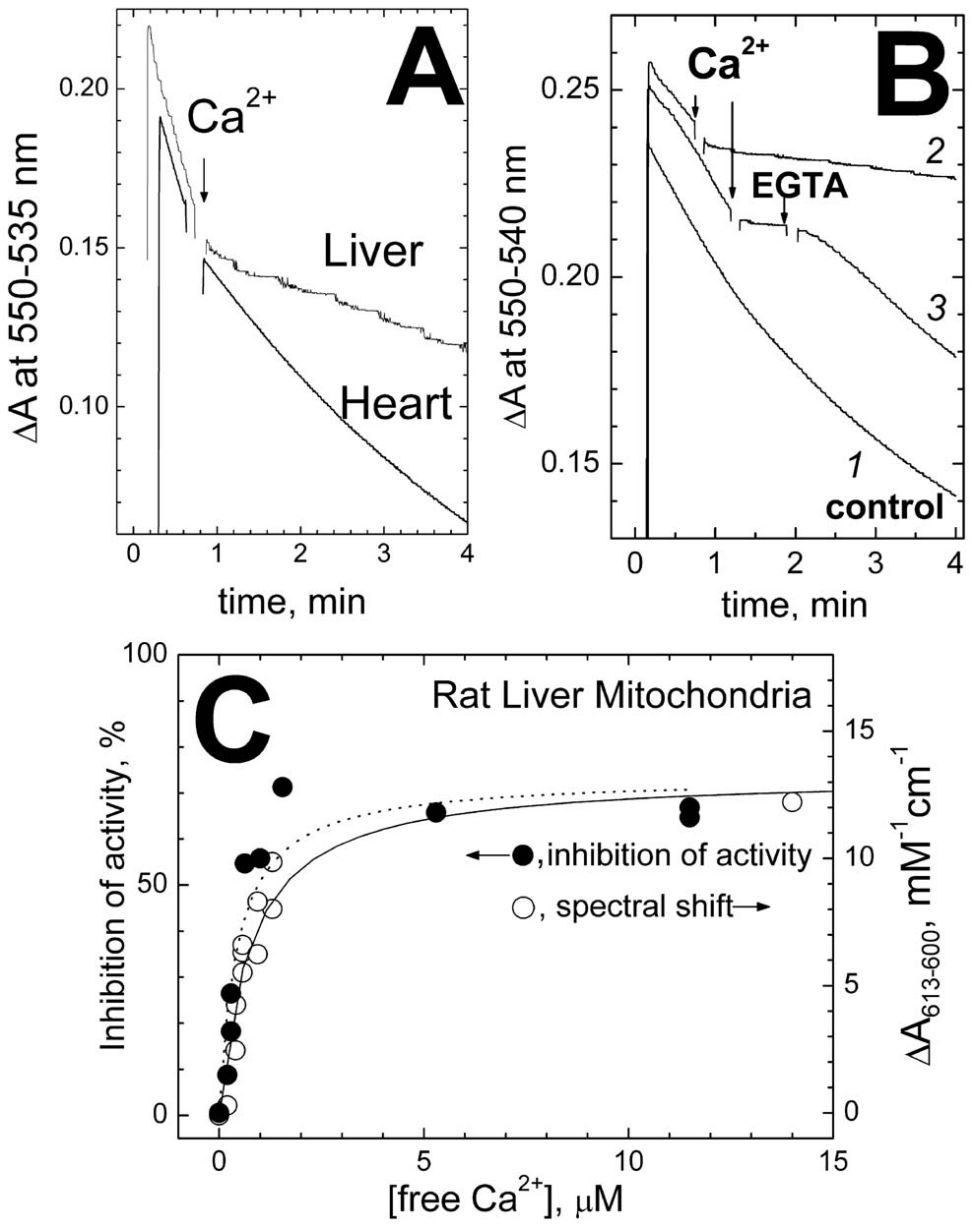
Ca^2+^-induced inhibition of ferrocytochrome *c* oxidase activity in mitochondria. (**A**) Rat liver (1.3 mg protein/ml) or rat heart mitochondria (0.6 mg protein/ml) in the medium containing 0.25 M sucrose, 50 mM HEPES/tris pH 8.0, 0.4 M choline chloride, 100 µM EGTA, and also 1 µM cyclosporine A and 1 µM the uncoupler, CCCP. 15 µM of the reduced cytochrome *c* is added and its oxidation is followed at 550 nm vs the 535 nm reference. Ca^2+^ addition, 200 µM. (**B**) Rat liver mitochondria. Conditions, as in trace *1* of panel **A**. Additions: CaCl_2_, 200 µM; EGTA, 300 µM. The traces in the Panels A,B have been displaced arbitrarily on the ordinate scale for clarity. (**C**) Concentration dependences of the Ca^2+^-induced inhibition of COX and spectral shift of heme *a* in rat liver mitochondria. Cytochrome *c* ^2+^ oxidation was measured as in **Figure 7B**, trace *1* at different concentrations of free calcium buffered with 5 mM HEDTA or 5 mM NTA. The initial slopes of the kinetic curves were used to build the plot. The data have been approximated by a hyperbolic curve (solid line) with the maximal inhibition of 74% and K_i_ = 0.76 µM. The Ca^2+^-induced spectral shift of the reduced heme *a* was measured in an SLM-Aminco-2000C spectrophotometer (see Materials and methods). To decrease light scattering, the mitochondria (10–15 mg protein/ml) were treated with 1% dodecyl maltoside. The data are approximated by a curve (dashed line) with ΔA_613–600_ (max) = 13 mM^−1^cm^−1^, and K_d_ = 0.56 µM.

As shown in **Figure 7A**, Ca^2+^ inhibits oxidation of ferrocytochrome *c* by rat heart mitochondria about 2-fold. Similar results were obtained for mitochondria isolated from bovine heart (the **Table**). Stronger inhibition is observed with mitochondria from rat liver **(Figure 7ABC**, the **Table**). The inhibition is reversed by addition of excess EGTA (**Figure 7B**, trace *3*). No inhibition was induced by Mg^2+^ (not shown). The inhibition of COX in liver mitochondria titrates with [I]_50_ of ∼0.5 µM (**Figure 3C**, filled circles). The concentration dependence appears to be slightly sigmoidal, but it can be approximated reasonably well by a hyperbolic curve with K_i_ of 0.75 µM and, within the experimental scatter, the data overlap with the titration of the Ca^2+^-induced red shift of heme *a* (open circles, K_d_ ∼0.5 µM). Thus, like in the case of the purified bovine heart oxidase, the inhibition of COX activity in rat liver mitochondria correlates with calcium binding at the site responsible for the spectral shift of heme *a.* As with the soluble cytochrome oxidase, no significant effect of Na^+^ on the activity could be observed, but sodium ions prevented the Ca^2+^-induced inhibition (data not shown).

The **Table** summarizes the inhibitory effects of Ca^2+^ on the COX activity of mitochondria from several rat tissues. The inhibition in mitochondria from the non-excitable tissues (liver and kidney) appears to be stronger than in the heart or skeletal muscle mitochondria. It is noted that the incomplete inhibition of COX by Ca^2+^ observed in the experiments may be explained, as usual, either by partial inhibition of the entire population of the enzyme or by complete inhibition of variable fraction of the enzyme (or both). More experiments are required to distinguish between these possibilities.

## Discussion

The principal finding of this work is that Ca^2+^ ions at concentrations of few µM inhibit mitochondrial cytochrome *c* oxidase, both in the soluble state and in the mitochondrial membrane. The inhibition is associated with Ca^2+^ binding at the special cation binding site of COX [10], [11] located at the outer face of the mitochondrial membrane (cf. **Figure 1**) and, peculiarly, is observed only at low turnover rates of the enzyme.

### Why was the Inhibition not Observed Earlier?

There are several probable reasons. First, the COX activity assays are usually aimed to reveal the maximal turnover rate of the enzyme. As found in this work, at high concentrations of the fully reduced ferrocytochrome *c* and turnover rate of ∼10^2^ s^−1^ or higher at pH 8, no inhibitory effect of Ca^2+^ is observed (e.g., **Figure 4A**). Second, the effect of Ca^2+^ is characterized by rather high affinity of the enzyme for the cation (K_i_ ∼ K_d_ ∼ 10^−6^ M). The reaction media used in experiments may easily contain some 5–10 µM of adventitious Ca^2+^ unless special precautions are taken. Therefore, the CBS of COX may be saturated with Ca^2+^ already before addition of exogenous calcium. In order to control free Ca^2+^ concentration in the µM range, Ca^2+^ buffers have to be used which was not often the case in the past works on Ca^2+^ interaction with COX (e.g. [12], [19], [40]). Third, Ca^2+^ binding with COX is antagonized by Na^+^, concentration of which in the buffers was rarely specifically controlled. Combination of these factors can readily explain why the inhibitory effect of Ca^2+^ on the cytochrome *c* oxidase activity was not noticed earlier.

As a matter of fact, scattered evidence for inhibition of the mitochondrial respiration by micromolar Ca^2+^ can be found in the literature (e.g., cf. Fig. 4 of ref. [27], Table 1 in ref. [41], ref. [42]), and the inhibition may be attributed, at least partly, to the inhibitory effect of the cation on the mitochondrial COX. (Take notice that the specific activity of the mitochondrial COX was determined in ref. [42] under high turnover conditions at which the inhibition of COX itself by Ca^2+^ would not be observed).

**Table 1:** Inhibition of the mitochondrial cytochrome *c* oxidase activity by Ca^2+^.

	Inhibition, %%			
	Heart	Skeletal muscle	Liver	Kidney
				
Rat mitochondria	50±5	60±5	80±2	76±4
Bovine mitochondria	57±4	–	–	–

Oxidation of 15 µM ferrocytochrome *c* by mitochondria from different tissues (0.5–1.5 mg protein/ml) was followed spectrophotometrically under low-turnover conditions as described in “Materials and Methods” and legend to **Figure 7**. The data correspond to inhibition induced by addition of excess Ca^2+^ (200 µM on the backgrpound of 100 µM EGTA). The data are average ± SE for 3–5 measurements.

### The Mechanism of the Ca^2+^-induced Inhibition of COX

Several possible explanations may be considered.

#### (i) Effect of Ca^2+^ on cytochrome c binding with COX

Binding of cytochrome *c* with COX is essentially electrostatic, so one could propose that Ca^2+^ competes with cytochrome *c* for the anionic binding site at Cu_A_. However, the observed inhibitory effect of Ca^2+^ requires the cation binding at the specific Cation Binding Site separate from the cytochrome *c* docking site; the effect is not mimicked by Mg^2+^ and is not observed with the bacterial oxidases. Furthermore, the inhibition of bovine oxidase by Ca^2+^ can be observed with the artificial electron donors - anionic ferrocyanide (**Figure 3A**) and uncharged TMPD (**Figure 3B**). Therefore, the explanation *(i)* is not likely.

#### (ii) Modulation of intraprotein electron transfer due to a shift of heme a midpoint redox potential

According to our initial hypothesis [43], the inhibition of COX by Ca^2+^ could be related to a positive shift of E_m_ of heme *a* induced by the cation [18], [29], [39]. However, we found that Ca^2+^ does not inhibit activity of mutant COX from *R. sphaeroides* (D485A) or *P.denitrificans* (D477A) (cf. **Figure 6**). These mutant oxidases are fully active and bind Ca^2+^ at the CBS reversibly in much the same way as bovine oxidase [14], [17], [18]. Moreover, a Ca^2+^-induced positive shift of E_m_ of heme *a* by 40–50 mV is observed with D477A oxidase from *P. denitrificans* that is stronger than the 15–20 mV shift found with the bovine COX ([39], paper in preparation). Nevertheless, there is no inhibition of the mutant D477A oxidase by calcium ions (**Figure 6**). Thus, the inhibitory effect of Ca^2+^ is not likely to be a simple consequence of ΔE_m_ of heme *a* and the inhibition may be specific for the mammalian COX.

#### (iii) Inhibition of H^+^ transfer through the proton conducting pathway H

**Figure 8:**
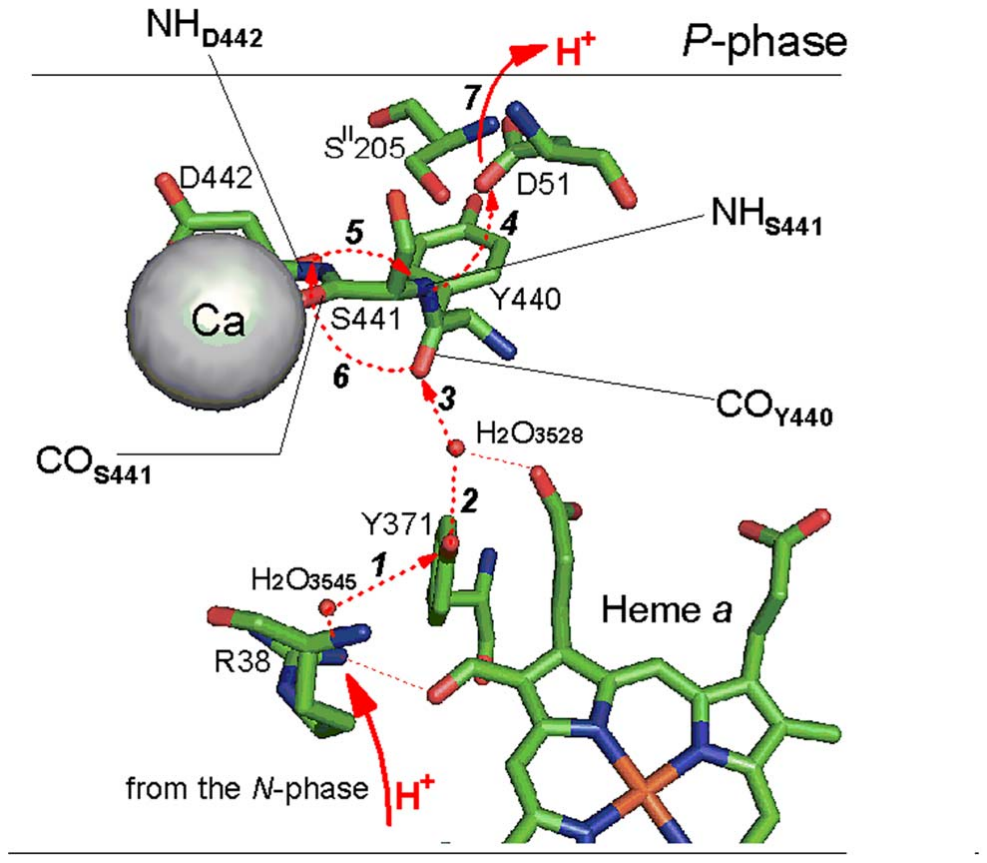
Interaction of Ca^2+^ with the exit of proton channel H in bovine heart oxidase. Structure of the exit part of the H-channel is shown based on the oxidized COX crystal structure, PDB entry 1V54. All the groups shown belong to subunit I (polypeptide A) except for S^II^205 from subunit II (polypeptide B). The scheme gives a scenario of proton transfer combined from refs. [44], [45], [49]. Proton trajectory is depicted by red arrows and sequence of the proton transfer steps is indicated by numbers. Oxidation of heme *a* by the binuclear site brings about a conformational change that unlocks the H-channel below the heme [46] (cf. [50] for an alternative proposal) and pumped proton arrives to the guanidine group of R38 from the *N*-aqueous phase (step ***1***) via the input part of the H-channel (cf. **Figure 1A**); it travels further via the R38-bound H_2_O_3545_ (that has then to shift closer to Y371), OH group of Y371 and H_2_O_3528_ to finally protonate the backbone carbonyl group of Y440 (steps ***2***–***4***). The carbonyl function C = O_Y440_ protonated, makes the -NH_S441_ acidic (imidic acid) and allows for its facile spontaneous deprotonation by carboxylate of D51 (step ***5***), converting the S441-Y440 peptide bond to the enol form. The protonated state of D51 is then stabilized by multiple hydrogen bonding to S^II^205 and S441. As proposed in [44], the enol form of the S441-Y440 peptide bond returns to the initial keto form (the notorious proton transfer via the peptide bond S441-Y440 [44], [45], [49]) actually in two steps. First, the deprotonated enolic = N_S441_ receives proton from the backbone -NH of D442 (step ***6***, the rate limiting stage of the entire process), which is followed by facile reprotonation of -N^-^_D442_ by the protonated backbone C-OH_Y440_ (step ***7***) returning the latter to the initial carbonyl state. Upon subsequent reduction of heme *a*, D51 undergoes reorientation associated with loss of the stabilizing hydrogen bonding to S^II^205 and, hence, decreased proton affinity, so that its carboxylic group releases the proton to the *P*-phase (step ***8***). Ca^2+^ coordinates to the backbone carbonyl oxygen of S441, and also makes a bond with the carboxylate of D442 via intercalated fixed water molecule (hidden behind the Ca ion in this projection of the structure, cf. H_2_O_3544_ in **Figure 1B** and see ref. [14]). As discussed in the text, Ca^2+^ is expected to inhibit proton transfer through the exit part of the H-channel and, accordingly, to impede the proton transfer-coupled electron transfer by COX.

As discussed in [15], [16], [18], [21], [44] the cation binding site in COX is adjacent to the exit part of the so-called “proton-conducting pathway H” or, simply, the “H-channel” described by Yoshikawa, Tsukihara and collaborates [11], [45] (**Figure 1A**, **Figure 8**). The H-channel has been proposed to be involved in translocation of the pumped protons by bovine heart COX and the proposal has received some experimental support [11], [45]–[50]. Alternative functions of the H-channel were considered in [15], [18], [51]. It was proposed that the channel may carry out controlled dissipation of proton gradient [15], [18] being involved in the so-called “mild uncoupling” of mitochondria [52], or function as a “dielectric channel” [51] facilitating electron transfer through heme *a*.

Crystal structure of the Ca^2+^-bound CBS in bovine COX has not been published yet, but it was modeled [17], [18] assuming similarity between coordination of Ca^2+^ and Na^+^ in bovine COX and taking into account the resolved structure of the Ca^2+^-loaded site in the bacterial oxidases (refs. [14], [15], PDB entry 1M56). Among the other groups, the cation is coordinated by the backbone carbonyl function of S441 [11], [44] (cf. **Figure 1B** **,** **Figure 8**). This serine located in a cytoplasmic loop connecting transmembrane helices XI and XII of subunit I is conserved in the oxidases from higher animals (annelid worms and higher) but is absent from COX of bacteria, yeast, fungi or higher plants (refs. [16], [53], bioinformatics search made in our group by N.Tretyakova). According to the proton pumping mechanism considered by Yoshikawa and collaborates [47], [54], S441 plays a key role in proton conduction through the exit part of the H-channel (**Figure 8**). First, its backbone amide group is proposed to be involved in delivery of the pumped proton to D51 via the peptide bond Y440-S441 [47], [48]. Second, the side chain of S441 makes a hydrogen bond to D51, a residue that gates release of the pumped H^+^ to cytoplasm changing its conformation upon reduction of heme *a* [11], [49]. Besides the interaction with S441, Ca^2+^ is likely to strongly interact with the carboxylate of D442 via intercalated fixed water [14], [18], whereas the amide function of D442 is proposed to participate in the two-step H^+^-transfer across the Y440-S441 peptide bond (**Figure 8**, ref. [44]).

Binding of Ca^2+^ at the CBS may be envisaged to inhibit proton transfer through the exit part of the H-channel. First, there are positively charged intermediates, such as hydronium cation H_3_O^+^_3528_ or the imidic acid state HOC = NH^+^ of the Y440-S441 peptide bond, that are postulated to be formed along the H^+^-transfer pathway through the exit part of the H-channel [44], [45], [49], [55]. The extra positive charge introduced by Ca^2+^ should hamper generation of such intermediates in vicinity of the cation. Second, and perhaps more important, the geometry of the bonds and distribution of the electronic clouds in the Y440-S441-D442 backbone segment are finely tuned in order to allow for the two-step mechanism of H^+^ transfer “through the Y440-S441 peptide bond” involving the Y440-S441 and S441-D442 peptide groups [44], [55]. Strong interactions of Ca^2+^ with the backbone carbonyl of S441 and with the side-chain of D442 are envisaged to distort the optimized structure and to make the site more rigid, thus raising the activation energy of the D442 backbone amide-assisted H^+^- transfer through the Y440-S441 peptide bond. Therefore, Ca^2+^ binding at the CBS is expected to inhibit intramolecular transfer of the protons through the exit of the H-channel and, accordingly, to hinder the redox activity of the enzyme coupled to the proton transfer.

It is tempting to speculate that in the bacterial A-class oxidases [13] in which the H-channel is partly conserved [47] but is not involved in proton pumping [56], [57], the tightly-bound calcium at the homologous CBS [10], [14], [15] may serve as a plug preventing backward proton leak through the idle H-pathway. The same role of locking securely the non-operative proton pathway can be proposed for the tightly-bound Ca^2+^ in the *cbb*_3_ oxidases, where the cation binds with the critical residues gating connection to the exit of the D-channel-associated proton pumping pathway that works in the A-type oxidases but is non-operative in the C-type *cbb*_3_ oxidases [58], [59].

### Why is the Ca^2+^-induced Inhibition Observed Only at Low Turnover Rates?

As the molecular mechanism of the calcium-induced inhibition of COX is not yet clear, there is no obvious answer to the question. In particular, it is difficult to discriminate between two interrelated factors: slow turnover per se and low steady-state reduction level of the redox centers in the enzyme. Preliminary experiments in which the rate of COX turnover was varied at a constant redox potential of cytochrome c (a 1∶1 mixture of the ferrous and ferric forms) indicate that the turnover rate may be itself important (**Figure S1**), but it does not exclude the role of redox potential of cytochrome *c*. Much more experiments are required to clarify the quantitative aspects of the Ca^2+^-induced inhibition. In any case, it is worth noting that, firstly, the low turnover rates at which the inhibition is observed most clearly (∼10 s^−1^ or less) are close to typical turnover rates of COX in the respiratory chain of mitochondria (ca. 100 ng-at O_2_ • min^−1^ • (mg protein) ^−1^, i.e. ∼10 s^−1^, assuming 0.3 nmol of *aa*_3_ per mg of protein of heart mitochondria [35]). Secondly, at oxygen concentrations above 1 µM, cytochrome *c* is only partly reduced in the actively respiring mitochondria (typically, 25–50% [35], [36]) as in our experiments, while it is almost fully reduced under the conditions of the standard COX assays. Therefore, the parameters of COX turnover under which the calcium inhibitory effect is observed are more relevant to physiological mode of the enzyme operation than those during the conventional conditions of COX activity assays. Different modes of COX operation at high- and low-turnover conditions in the cell have been discussed in the literature (reviewed, [60]–[63]) and the oxidase that turns over slowly is more susceptible to regulation by nucleotides similarly to modulation of the activity by calcium ions in this work. Higher susceptibility of COX to inhibition under low-turnover conditions has been described also for the inhibitory effect of Zn^2+^ ions [40], [64], [65]. Interestingly, S441 is located in a sequence RRYS that is a canonical target for protein kinase A, which may be a one more interesting aspect of the regulatory function of the CBS [15], [53], [60].

### Physiological Significance of the Ca^2+^-induced Inhibition of COX

It remains an open question, to which extent the Ca^2+^-induced inhibition of COX described in this work may be involved in regulation of oxidative phosphorylation by the cation under physiological conditions. Typical cytoplasmic concentration of free Ca^2+^ under resting conditions is 0.1–0.2 µM [23], i.e. below K_d_ ∼1 µM of Ca^2+^ binding with COX. Moreover, if competition of Ca^2+^ with Na^+^ is taken into account [16], [20], then at 5–10 mM of cytoplasmic Na^+^ [66] the effective K_d_ for Ca^2+^ binding with COX should rise to ∼10^−5 ^M [16], [18]. Therefore under the resting conditions, the cation binding site of COX is expected to be occupied by Na^+^ (K_d_ ∼ 10^−3^ M [16]) that does not inhibit the enzyme. However, during release of Ca^2+^ from endoplasmic reticulum or upon its entry from the extracellular stores in response to various stimuli, local concentrations of Ca^2+^ in microdomains near mitochondria “can readily reach many tens of micromolar” [67], [68] or even 100 µM [69], so that transient displacement of Na^+^ and binding of Ca^2+^ to COX during the spikes may well take place. The major potential consequences of such transient binding could be as follows.

### (i) Inhibition of the Mitochondrial Respiration

Ca^2+^ enhances oxidative phosphorylation as it is taken up inside mitochondria and activates there several Krebs cycle dehydrogenases (reviewed, [24], [25], [70]) and perhaps some respiratory chain cytochrome complexes [71]. Ca^2+^ also acts from the outside of mitochondria by activating the *aralar-* and *citrin* -type substrate transporters in mitochondria from some tissues [26], [27], [70]. Our data indicate that in addition, the cytoplasmic (extramitochondrial) Ca^2+^ ions may slow down mitochondrial respiration by direct inhibition of cytochrome *c* oxidase (cf. [27], [41], [42]).*(ii) Stimulation of Reactive Oxygen Species (ROS) production.* Activation of the dehydrogenases and concomitant inhibition of COX should act synergistically in increasing the reduction of the respiratory chain carriers at given NAD(P)H/NAD(P)^+^ and GSH/GSSG ratios in the mitochondrial matrix. In this way Ca^2+^ is expected to stimulate ROS production by the respiratory chain (cf. [72] and refs. therein). Transient increase in ROS production may be in its turn part of intracellular signal transmission cascade in response to various stimuli.

*(iii) Modulation of calcium uptake by mitochondria.* Ca^2+^ is taken up actively by respiring mitochondria via the so-called calcium uniporter, a highly specific Ca^2+^ channel, down the Δψ formed by the respiratory chain ([66], [73], [74] and refs. therein). Inhibition of COX and hence of the entire respiratory chain by Ca^2+^ is expected to slow down the respiration-driven Ca^2+^ uptake and to attenuate accumulation of Ca^2+^ in the mitochondrial matrix. It is noted that at cytoplasmic concentrations of Na^+^, affinity of COX for Ca^2+^ virtually coincide with the effective “K_m_” of the mitochondrial calcium uniporter (ca. 10 µM [74]). Therefore, cytochrome oxidase and the calcium uniporter are expected to respond to changes in cytoplasmic [Ca^2+^] within the same concentration range, but perhaps with different time constants, as there is a significant lag in activation of the calcium uniporter. This may be an interesting object for modeling.

In the excitable tissues like heart or skeletal muscle, the Ca^2+^-induced inhibition of COX is moderate (∼2-fold). A likely role of the inhibition might consist in damping the effects of the periodical [Ca^2+^_free_] spikes in the cytoplasm [68], [70]; such damping would protect mitochondria from overloading with Ca^2+^ during the spikes. In the non-excitable tissues, mitochondria that are able to suck in rapidly calcium released to the cytoplasm from the endoplasmic reticulum cisterns or delivered from the extracellular stores, are active players in the Ca^2+^ -mediated signal transmission and participate in “shaping in time and space” the cytoplasmic Ca^2+^ signals [23], [75], [76]. Significant inhibition of COX in liver or kidney mitochondria by Ca^2+^ could then be involved in modulation of the multiple regulatory pathways in which mitochondria interfere with the cellular response to Ca^2+^.

## Conclusions

1. The direct inhibitory effect of Ca^2+^ on mammalian cytochrome oxidase is an important novel factor potentially involved in regulation of oxidative phosphorylation, mitochondrial ROS production and intracellular transmission of the calcium signals.

2. Location of the calcium binding site near the exit of the so-called proton conducting pathway H along with the specific inhibitory effect of Ca^2+^ on the mammalian, but not on the bacterial, cytochrome oxidases, favour functional significance of the “H-channel” in cytochrome oxidase from mammalian mitochondria. Ca^2+^ may be proposed to be a physiological specific inhibitor of the H-channel, whatever the function of the channel is.

## Supporting Information

Figure S1 The calcium-induced inhibition of cytochrome c oxidase at different concentrations but constant redox potential of cytochrome *c*. The reaction mixture contained 50 mM tris-MES buffer, pH 8, with 0.4 M choline chloride, 0.05% DM, 100 µM EGTA and 1∶1 mixture of the oxidized and reduced forms of cytochrome *c* at concentrations indicated (1–55 µM of each). The cytochrome oxidase reaction was initiated by addition of 6 nM purified bovine COX, and oxidation of cytochrome *c* ^2+^ was followed spectrophotometrically in a dual-wavelength mode at 550 nm vs the 535 nm reference. Where indicated, 200 µM Ca^2+^ (100 µM excess over EGTA) was also present. The curves are drawn through the points to guide the eye. *Is turnover rate an essential factor that determines cytochrome c oxidase sensitivity to inhibition by Ca^2+^?* In a pilot experiment shown in **Figure S1**, concentration of cytochrome *c* was varied to change the cytochrome *c* oxidase reaction rate, while the redox potential was kept constant by using equimolar mixture of the reduced and oxidized cytochrome *c* for each point. It can be seen that the curves in the absence and in the presence of calcium tend to converge as the reaction rate increases. For instance, the inhibition induced by Ca^2+^ is 57% at the lowest rate of electron transfer (1.2 s^−1^), 44% at 6.1 s^−1^ and 21% at 12 s^−1^. In agreement with the data in **Figure 4A**, no inhibition at all was observed at turnover rate of 105 s^−1^; the latter point (not included in **Figure S1**) was measured with this sample of COX in a separate experiment with 15 µM ferrocytochrome as the electron donor but without ferric cytochrome *c* and at choline concentration of 50 mM. Results of the experiment confirm that the turnover rate is indeed an essential factor affecting the sensitivity of COX to inhibition by calcium. Conceivably, the data do not exclude contribution of other factors, such as redox potential. It is noted that the dependencies in are to be considered as preliminary data and apply just to one of the several COX samples studied in this work. The exact profile for the Ca^2+^-induced inhibition as a function of turnover rate may vary for different preparations. (E.g., in the preparation for which most of the data presented in the paper have been obtained, inhibition of 58±2% was observed at turnover rate of 6 s^−1^, rather than 44% as in **Figure S1)**. As we do not know yet all the parameters affecting the Ca^2+^-sensitivity of COX, much more experiments are required for quantitative description of the phenomenon.
(TIF)
